# Lethal Antibody Enhancement of Dengue Disease in Mice Is Prevented by Fc Modification

**DOI:** 10.1371/journal.ppat.1000790

**Published:** 2010-02-12

**Authors:** Scott J. Balsitis, Katherine L. Williams, Ruben Lachica, Diana Flores, Jennifer L. Kyle, Erin Mehlhop, Syd Johnson, Michael S. Diamond, P. Robert Beatty, Eva Harris

**Affiliations:** 1 Division of Infectious Diseases and Vaccinology, School of Public Health, University of California, Berkeley, Berkeley, California, United States of America; 2 Department of Pathology & Immunology, Washington University School of Medicine, St. Louis, Missouri, United States of America; 3 MacroGenics, Inc., Rockville, Maryland, United States of America; 4 Department of Molecular Microbiology, Washington University School of Medicine, St. Louis, Missouri, United States of America; 5 Department of Medicine, Washington University School of Medicine, St. Louis, Missouri, United States of America; 6 Department of Molecular and Cellular Biology, University of California, Berkeley, Berkeley, California, United States of America; University of North Carolina, United States of America

## Abstract

Immunity to one of the four dengue virus (DV) serotypes can increase disease severity in humans upon subsequent infection with another DV serotype. Serotype cross-reactive antibodies facilitate DV infection of myeloid cells *in vitro* by promoting virus entry via Fcγ receptors (FcγR), a process known as antibody-dependent enhancement (ADE). However, despite decades of investigation, no *in vivo* model for antibody enhancement of dengue disease severity has been described. Analogous to human infants who receive anti-DV antibodies by transplacental transfer and develop severe dengue disease during primary infection, we show here that passive administration of anti-DV antibodies is sufficient to enhance DV infection and disease in mice using both mouse-adapted and clinical DV isolates. Antibody-enhanced lethal disease featured many of the hallmarks of severe dengue disease in humans, including thrombocytopenia, vascular leakage, elevated serum cytokine levels, and increased systemic viral burden in serum and tissue phagocytes. Passive transfer of a high dose of serotype-specific antibodies eliminated viremia, but lower doses of these antibodies or cross-reactive polyclonal or monoclonal antibodies all enhanced disease *in vivo* even when antibody levels were neutralizing *in vitro*. In contrast, a genetically engineered antibody variant (E60-N297Q) that cannot bind FcγR exhibited prophylactic and therapeutic efficacy against ADE-induced lethal challenge. These observations provide insight into the pathogenesis of antibody-enhanced dengue disease and identify a novel strategy for the design of therapeutic antibodies against dengue.

## Author Summary

Dengue is the most common vector-borne viral disease of humans, with over 3 billion people at risk for infection and 50–100 million infections in tropical and subtropical regions each year. Dengue virus (DV) causes a spectrum of clinical disease ranging from an acute debilitating, self-limited febrile illness (DF) to a life-threatening vascular leakage syndrome, referred to as dengue hemorrhagic fever/dengue shock syndrome (DHF/DSS). There are four serotypes of DENV; infection with one serotype is thought to protect against re-infection with the same serotype, but may either protect against or enhance infection with one of the other three serotypes. Epidemiological and *in vitro* data has implicated anti-DENV antibodies in mediating pathogenesis of a second DENV infection. However, it is unclear which antibody conditions are protective and which exacerbate disease *in vivo*, in part because no animal model of antibody-enhanced dengue disease has been available. Here, we present the first animal model of antibody-enhanced severe DENV infection. Importantly, this model recapitulates many aspects of human disease, including vascular leakage, elevated serum cytokine levels, reduced platelet count, and disseminated infection of tissue phagocytes. Furthermore, we demonstrate the utility of this model by showing that a genetically modified anti-DENV antibody that fails to bind the Fcγ receptor has prophylactic and therapeutic efficacy against lethal DENV challenge *in vivo.*

## Introduction

The four serotypes of dengue virus (DV) are mosquito-borne flaviviruses responsible for 50–100 million human infections annually. Primary infection in individuals over the age of one year with any DV serotype is usually asymptomatic or results in self-limited dengue fever (DF), but secondary infection with another DV serotype carries an increased risk of severe disease, including life-threatening dengue hemorrhagic fever/dengue shock syndrome (DHF/DSS) [1],[2]. Fatal disease is characterized by increased vascular permeability leading to hemoconcentration and hypovolemic shock [3]. The increased severity of secondary infections is believed to result, at least in part, from antibody-dependent enhancement (ADE) of DV infection, in which FcγR engagement by antibody-virus immune complexes facilitates virus entry into susceptible myeloid cell types [4]. A role for ADE in human dengue pathogenesis is supported by observations that maternally-derived anti-DV antibodies increase the risk of DHF in infants during primary infection with DENV2 [5],[6]. Antibody-mediated increases in DV viremia have been demonstrated in macaques, but a limited number of antibody conditions were examined, and exacerbation of dengue disease by passively transferred antibodies was not observed [7],[8]. Consequently, fundamental questions about the immunology and pathogenesis of ADE have remained unanswered, and small animal models for testing antiviral interventions in the context of ADE have not been available.

Recently, we derived a mouse-adapted DV2 strain, D2S10, that produces a TNF-α-dependent fatal vascular permeability syndrome in interferon-α/β and γ-receptor-deficient (AG129) mice 4–5 days after intravenous (iv) infection with 10^7^ plaque forming units (pfu) of virus [9]. DV infection in AG129 mice reproduces important features of human DV infection, including similar tissue and cellular tropism, viremia, vascular leakage, and elevated serum cytokine levels [9]–[12]. Antibodies elicited by DV infection are a mixture of serotype-specific and serotype-cross-reactive antibodies, including long-lasting neutralizing antibodies [13]. Memory immune responses are present after primary DV infection, and serotype cross-protective immunity was observed in three different sequential infection scenarios ([13] and data not shown). Thus, we utilized the AG129 model to examine the effects of serotype cross-reactive antibodies on DV2 infection *in vivo*. In this report, we demonstrate lethal enhancement of DV infection and disease by both polyclonal and monoclonal antibodies. We also show that ADE functions to increase the viral burden in blood and tissues, resulting in a vascular permeability syndrome that is similar to that seen in mice with a higher inoculum in the absence of immune antibody and that shares clinical features of human dengue disease. Finally, we confirm the critical role of FcγR interaction in ADE *in vivo* and provide proof-of-principle for a pre- and post-exposure treatment strategy utilizing genetically engineered monoclonal antibodies that can no longer bind FcγR.

## Results

### Lethal enhancement of dengue disease by anti-DV serum

**Figure 1:**
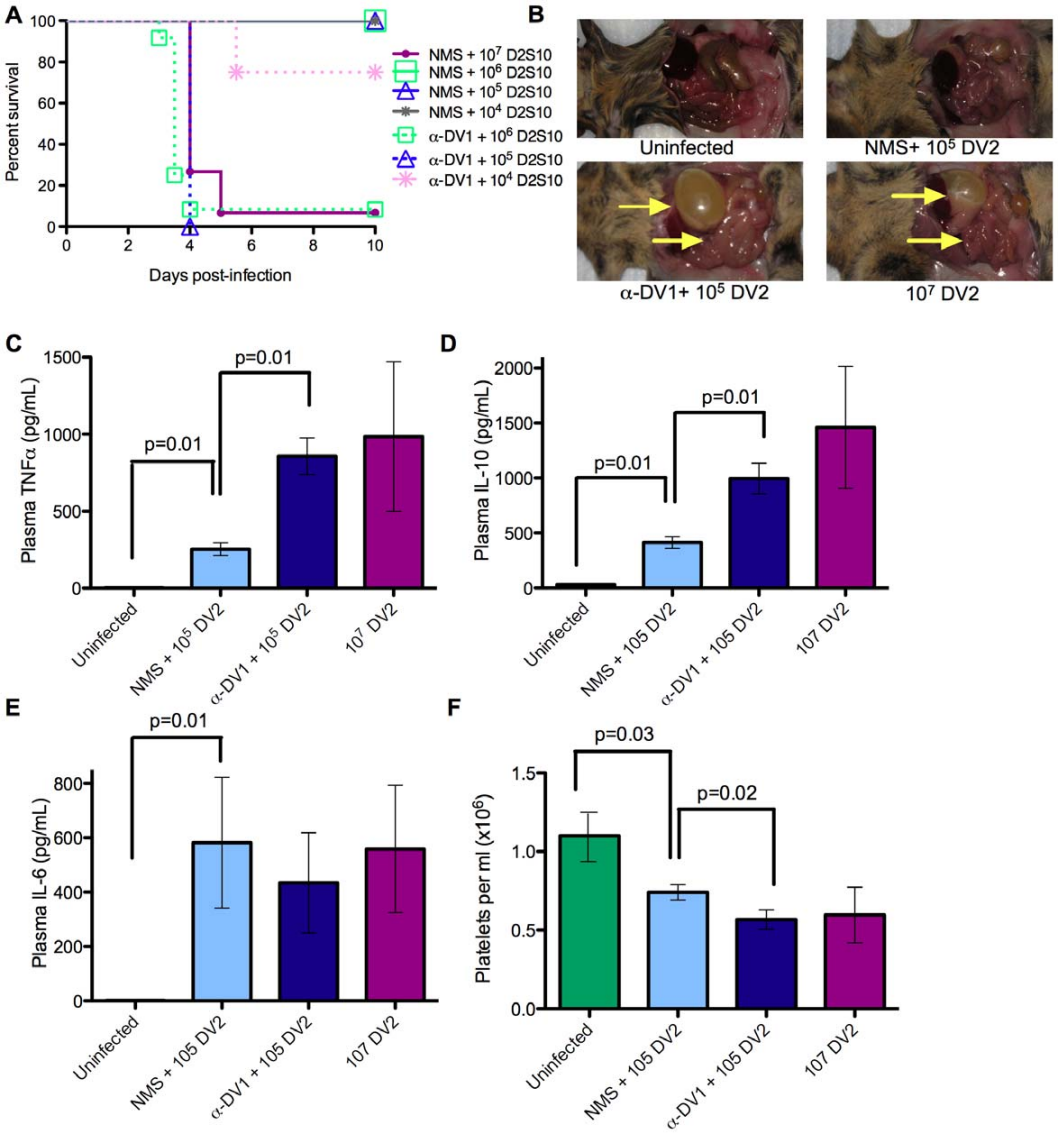
Lethal enhancement of dengue disease by anti-DV sera. **A.** Mice were administered 100 µl naïve mouse serum (NMS) or anti-DV1 serum (α-DV1), challenged iv with the indicated dose of DV2 strain D2S10 24 hours later, and monitored for survival. Kaplan-Meier survival curves are shown; see Table S1 for p-values. The numbers of mice per group are as follows: NMS +10^7^ D2S10, 15; NMS +10^6^ D2S10, 8; NMS +10^5^ D2S10, 11; NMS +10^4^ D2S10, 4; α98J +10^6^ D2S10, 12; α98J +10^5^ D2S10, 4; α98J +10^4^ D2S10, 4. **B–F.** Disease parameters were compared in mice receiving no virus, 10^7^ pfu DV2, or 10^5^ pfu DV2 after transfer of naïve or anti-DV1 serum. **B.** Vascular leak-associated fluid accumulation in visceral organs at day 3.5 post-infection. **C–E.** Serum levels of TNF-α (C), IL-10 (D), and IL-6 (E) in infected mice at day 3.5 post-infection were measured by ELISA (eBioscience). **F.** Platelet counts in mice at day 3.5 post-infection were determined using a hemocytometer. In (C–F), n = 4 in all groups except the uninfected group in panel F, where n = 12. Error bars represent standard deviations, and two-sided Wilcoxon rank sum tests were used to determine statistical significance.

Serum containing anti-DV1 antibodies was collected from AG129 mice 8 weeks after subcutaneous inoculation with 10^5^ pfu of DV1 strain 98J. Heat-inactivated anti-DV1 serum exhibited a 50% neutralizing titer (NT_50_) against DV2 strain D2S10 of 1∶296 and against DV1 98J of 1∶1,069 using a flow-based neutralization assay [14], peak enhancement titers of 1∶75 against DV2 D2S10 (fold-enhancement 14.8%) and 1∶225 against DV1 98J (fold-enhancement 10.7%) in an *in vitro* ADE assay with FcγR-bearing human K562 cells, and ELISA titers of 400 and 3200 against purified DV2 and DV1, respectively (data not shown). In addition, no residual DV1 could be isolated following inoculation into C6/36 mosquito cells (data not shown). The effects of anti-DV1 serum on DV2 infection were investigated after intraperitoneal (ip) injection of 100 µl of either naïve mouse serum (NMS) or anti-DV1 serum, followed 24 hours later by iv challenge with 10^4^–10^6^ pfu of DV2. Lethal infection controls received 10^7^ pfu of DV2, and all mice were monitored for mortality for 10 days. While no mortality was observed in NMS-recipient mice infected with 10^6^ pfu or less of DV2, 92–100% of anti-DV1 recipients died after inoculation with 10^5^–10^6^ pfu of DV2 (**Figure 1A** and **Table S1**) between 4 and 5 days post-infection. In both the 10^7^ pfu infection controls and anti-DV1 recipients infected with 10^5^ or 10^6^ pfu, lethal disease was accompanied by fluid accumulation in visceral organs characteristic of the vascular permeability syndrome induced by DV2 D2S10 [9] (**Figure 1B**). Mice administered anti-DV1 serum and challenged with DV2 D2S10 also experienced significant increases in serum TNF-α (*p*<0.01) and IL-10 (*p*<0.01) and greater platelet depletion (*p*<0.02), as compared to NMS-recipient controls (**Figure 1C–F**); each of these disease parameters also correlates with dengue severity in humans [15]–[17].

### Anti-DV antibody increases systemic viral burden

**Figure 2:**
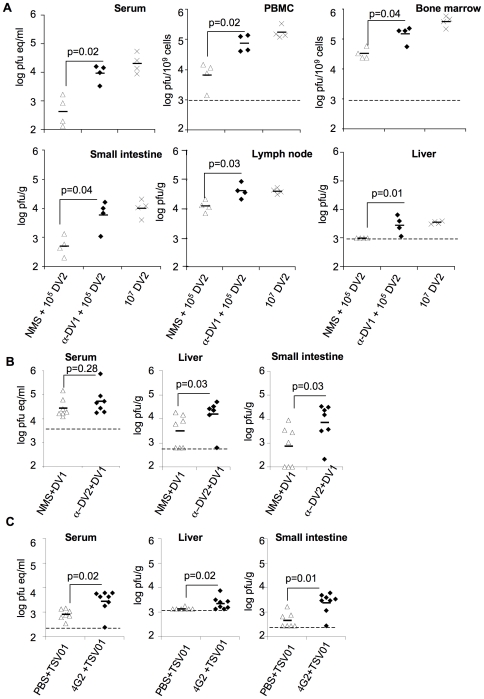
Enhancement by heterologous anti-DV antibodies increases systemic viral burden of mouse-adapted and clinical isolates of DV. **A.** Mice were administered 100 µl NMS or anti-DV1 serum ip and infected 24 hours later with 10^5^ pfu DV2 D2S10 iv; lethal infection controls were infected iv with 10^7^ pfu DV2 D2S10. Viral burden was measured in the indicated tissues at day 3.5 post-infection by qRT-PCR (serum) or plaque assay (all other tissues) as described in Materials and Methods. **B.** Mice were administered 100 µl NMS or anti-DV2 serum ip and infected the next day with 3×10^6^ pfu DV1 Western Pacific-74 iv. Virus burden was measured in the indicated tissues at day 3.5 post-infection by qRT-PCR (serum) or plaque assay (all other tissues) as described in the Materials and Methods **C.** Mice were administered 20 µg anti-DV MAb 4G2 or PBS ip and infected the next day with 10^6^ pfu DV2 TSV01 iv. Virus burden was measured as in (A). Symbols indicate values in individual mice. Limits of detection are represented by dashed lines when present, or the horizontal axes. All pairwise comparisons were performed by two-sided Wilcoxon Rank Sum tests.

Viral burden was subsequently compared between anti-DV1 and NMS-recipient mice infected with 10^5^ or 10^6^ pfu of DV2. Viral burden was systemically increased in anti-DV1 versus NMS-recipient mice, with a 20-fold increase (*p*<0.02) in viremia accompanied by significant 3- to 12-fold increases in viral load in multiple tissues (*p*≤0.04) including peripheral blood mononuclear cells, liver, small intestine, lymph node, and bone marrow (**Figure 2A**); non-significant increases in the large intestine and spleen (*p*>0.08) and lungs (data not shown) were observed. No statistically significant differences were observed in tested disease parameters, viral burden, or tissue tropism between 10^7^ pfu of D2S10 infection in the absence of antibody and antibody-enhanced infection with 10^5^ pfu of D2S10. Notably, anti-DV antibodies also enhanced infection with non-adapted low-passage human DV isolates DV1 Western Pacific-74 (**Figure 2B**) and DV2 TSV01 (**Figure 2C**), as determined by significant increases (*p*≤0.04) in viral burden in the liver and small intestine for both viruses, and in serum for DV2 TSV01. Although mortality was not observed, a subset of animals infected with DV1 Western Pacific-74 under antibody-enhanced conditions displayed fluid accumulation in visceral organs and gross morphology similar to but less pronounced than that observed with enhanced DV2 D2S10 disease.

### Increased infection of FcγR-bearing cells during ADE

**Figure 3:**
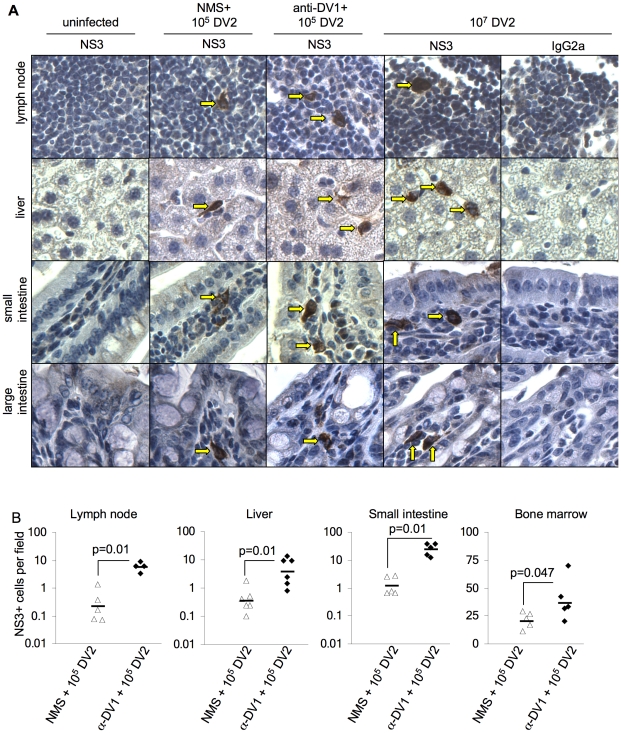
Detection and quantification of DV-infected cells with or without antibody-dependent enhancement. Mice were administered naïve serum (NMS) or anti-DV1 serum and infected iv with 10^5^ pfu DV2 the following day. Controls were mock-infected or infected with 10^7^ pfu DV2 iv. **A.** Tissues were collected from all mice (n = 3–6 per group) at day 3.5, formalin-fixed, and processed into paraffin sections. Serial sections from each tissue were stained with anti-DV NS3 antibody E1D8 (NS3) or an isotype control mouse IgG2a (IgG2a data not shown), and multiple sections of each tissue type were thoroughly examined for staining. Positive staining for NS3 is brown while hematoxylin counterstain is blue. Strong cytoplasmic staining observed with E1D8, but not IgG2a control antibody, was considered DV-specific when observed in infected mice but not uninfected controls. NS3^+^ cells in lymph node, small intestine, and large intestine exhibited morphology and location consistent with tissue macrophages under all infection conditions (arrowheads). In liver, NS3^+^ cells were consistent with tissue macrophages and/or endothelial cells. Serial sections showed the F4/80 macrophage marker staining in the same locations where infected cells were present in lymph nodes, small intestine, large intestine, and bone marrow (data not shown). **B.** NS3^+^ cells per visual field were quantified. At least ten visual fields were counted for each sample except bone marrow, where four fields from four independent sections were counted due to the small area of mouse bone cross-sections. All pairwise comparisons were performed by two-sided Wilcoxon Rank Sum tests.

ADE is predicted to facilitate infection of FcγR-bearing cell types such as tissue macrophages and dendritic cells [4]; therefore, we examined the cellular tropism of DV2 in mice by immunostaining for the viral NS3 protein, which is only present during active replication of the virus. As found in humans [12],[18], infected cells with morphology and location consistent with tissue macrophages or dendritic cells [19],[20] were detected in lymph node, small intestine, large intestine, and bone marrow under all infection conditions, and NS3^+^ cells with endothelial and/or phagocyte morphology were also observed in liver (**Figure 3** and data not shown). Infection in myeloid cells was confirmed by co-staining of serial sections and bone marrow aspirates for NS3 and the myeloid/macrophage marker F4/80 (data not shown). Furthermore, using flow cytometry, DV NS3 and E protein were detected in bone marrow cells expressing myeloid markers CD11b, CD11c, and F4/80, and in liver DV infection was primarily in CD31^+^CD45^−^ sinusoidal endothelial cells, which also express FcγR (**Figure S1**). Notably, significantly greater numbers of NS3^+^ cells (*p*≤0.05) were present in tissues of anti-DV1 recipient mice compared to naïve serum recipient controls infected with the same dose of DV2 (**Figure 3B**), supporting the hypothesis that ADE functions to increase the viral burden in cells and tissues.

### Effect of antibody dose on ADE *in vivo*

**Figure 4:**
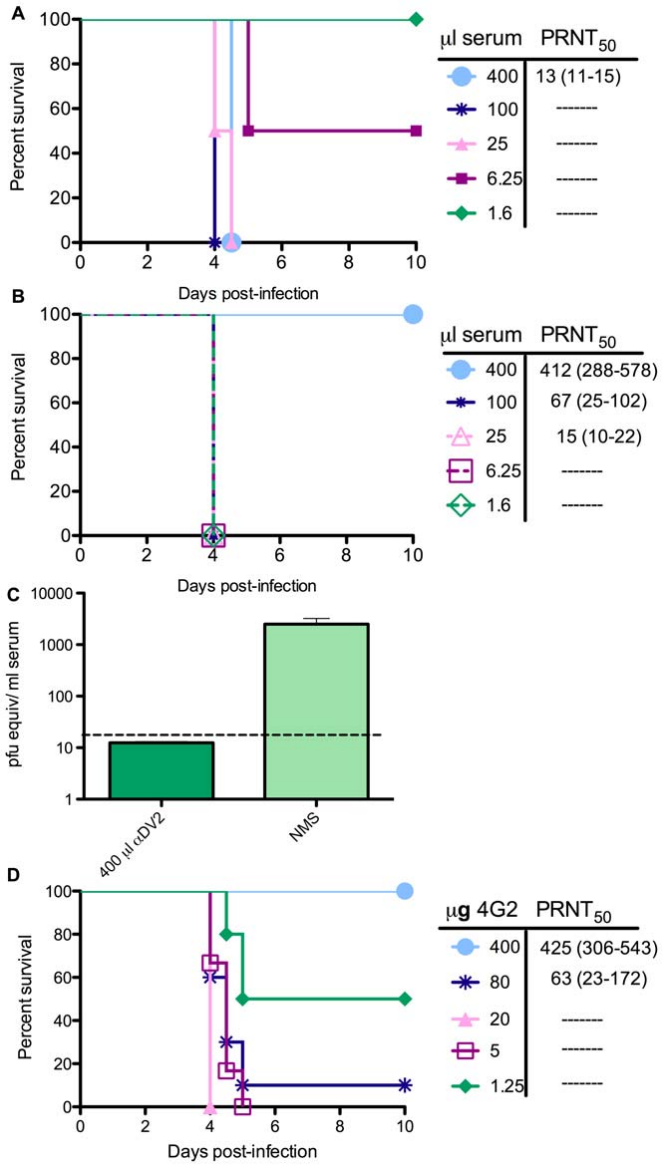
Antibody conditions for enhancement of DV infection. **A.** Mice were administered 1.6–400 µl anti-DV1 serum ip, and pre-infection serum samples were collected the next day. Mice were then infected with 10^5^ pfu DV2 iv and monitored for survival. Neutralizing activity of each pre-infection serum was determined in duplicate by PRNT_50_ assay on BHK21-15 cells. For each serum dose, PRNT_50_ results are displayed as the average of 3 to 4 mice, with the range in brackets. **B.** Serum transfers, bleeds, virus challenges, and PRNT assays were performed as in (A), but using anti-DV2 serum generated in AG129 mice. **C.** Viremia was measured in naïve serum controls (n = 4) and recipients of 400 µl anti-DV2 serum (n = 3) on day 4 post-infection by qRT-PCR. **D.** Mice were administered 1.25–400 µg of anti-DV monoclonal antibody 4G2, and pre-infection bleeds, challenges, and PRNT assays were performed as in (A).

While serotype cross-reactive immunity is implicated in the pathogenesis of severe dengue, serotype-specific immunity typically protects against re-infection with the same DV serotype [1]. However, *in vitro* studies suggest that all antibodies that neutralize infection are capable of ADE at some lower concentration [21]; therefore, we examined the effects of anti-DV1 and anti-DV2 sera on DV2 D2S10 infection in mice over a range of doses. While the highest dose (400 µl) of anti-DV1 serum lethally enhanced infection (**Figure 4A** and **Table S2**), recipients of 400 µl of anti-DV2 serum developed no signs of illness and lacked detectable viremia (**Figure 4B and C**; **Table S2**), confirming that serotype-specific antibodies can provide robust protection in this model. However, lower doses of both anti-DV1 and anti-DV2 serum caused lethal enhancement, showing that serotype-specific as well as serotype-cross-reactive antibodies can also enhance infection *in vivo* in a dose-dependent manner (**Figure 4A and B**). To assess the ability of the BHK PRNT_50_ assay to predict *in vivo* protection and enhancement in this mouse model, neutralizing activity was measured in the sera of mice immediately prior to infection with D2S10. Serum was collected approximately 18 hours post-transfer of anti-DV antibodies, and 4 hours prior to infection. Similar to human studies [22], lethal enhancement occurred even in mice that had detectable neutralizing antibodies, although no lethality was observed in mice with PRNT_50_ values of >200.

To further define the characteristics of enhancing antibodies, we examined the ability of monoclonal antibodies (mAbs) to enhance DV disease in mice. Mice were inoculated with DV2 D2S10 24 hours after transfer of increasing amounts of the flavivirus cross-reactive, neutralizing mAb 4G2 (**Figure 4D**). 4G2 caused lethal enhancement at doses of 0.062–4 mg/kg (1.25–80 µg/mouse), but no mortality occurred in mice receiving 20mg/kg (400 µg/mouse) or in IgG2a isotype control antibody recipients (**Figure 4D** and **Table S2**). 4G2, anti-DV1 serum, and anti-DV2 serum all enhanced infection and disease over a ∼60-fold range in concentration.

### ADE requires FcγR interaction *in vitro* and *in vivo*

**Figure 5:**
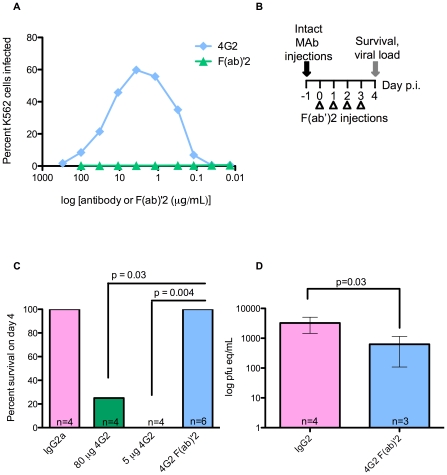
Antibodies that lack the Fc region fail to mediate ADE and instead protect against lethal challenge. **A.** Infection of K562 cells by DV2 in the presence of 4G2 mAb or 4G2 F(ab′)2 fragment was determined 48 hours post-infection by staining with Alexa488 anti-DV E protein MAb followed by flow cytometry. Average infection without antibody was 0.74%. **B.** Dosing scheme used to compare the in vivo effects of F(ab′)2 and intact mAb. Mice were administered intact 4G2 or IgG2a control mAb on day −1, or 20 µg doses of F(ab′)2 every 24 hours beginning 1 hour prior to infection, and were then challenged with 10^5^ pfu of DV2 iv. **C.** Survival in mice from (B) receiving the indicated antibodies was scored on day 4 post-infection, and a two-sided Fisher's exact test was used. **D.** Viremia at day 4 post-infection in surviving mice from (C), measured by qRT-PCR. Error bars represent standard deviations, and pairwise comparisons were performed by two-sided Wilcoxon rank sum tests.

Since FcγR engagement is required for ADE *in vitro* [23], we hypothesized that eliminating the ability of antibodies to bind to FcγRs would prevent ADE *in vivo*. To test this, we first generated F(ab′)2 fragments of 4G2. These fragments were indistinguishable from intact 4G2 in their ability to bind to DV2 E protein by ELISA (**Figure S2A**), but were unable to enhance DV infection of the human FcγR-bearing cell line K562 (**Figure 5A**). The lack of the Fc portion in the F(ab′)2 fragments of 4G2 was confirmed by ELISA (**Figure S2B**). *In vivo*, F(ab′)2 fragments have a shorter serum half-life than intact IgG, thus it was necessary to identify a dosing regimen that would maintain serum concentration of F(ab′)2 fragments within the known enhancing range for intact IgG antibodies. Serum F(ab′)2 levels were measured one and 24 hours after iv transfer of 20 µg of F(ab′)2 by E protein ELISA; this dose maintains E-reactive antibodies at levels within the range where IgG causes enhancement for 24 hours (**Figure S2C**). To examine the effects of intact IgG and F(ab′)2 *in vivo*, we compared the enhancing effects of a single dose of 4G2 mAb with daily 20 µg doses of 4G2 F(ab′)2 (**Figure 5B**). Whereas significant mortality was observed in 4G2 mAb recipients (*p*≤0.04), no illness occurred in 4G2 F(ab′)2 or IgG2a isotype control recipients (**Figure 5C**). Viremia measured at 3.5 days post-infection in F(ab′)2 recipients was significantly reduced (*p*<0.03) compared to isotype control antibody recipients (**Figure 5D**), suggesting that loss of FcγR interaction not only diminished enhancement but also promoted neutralization to reduce viral load.

**Figure 6:**
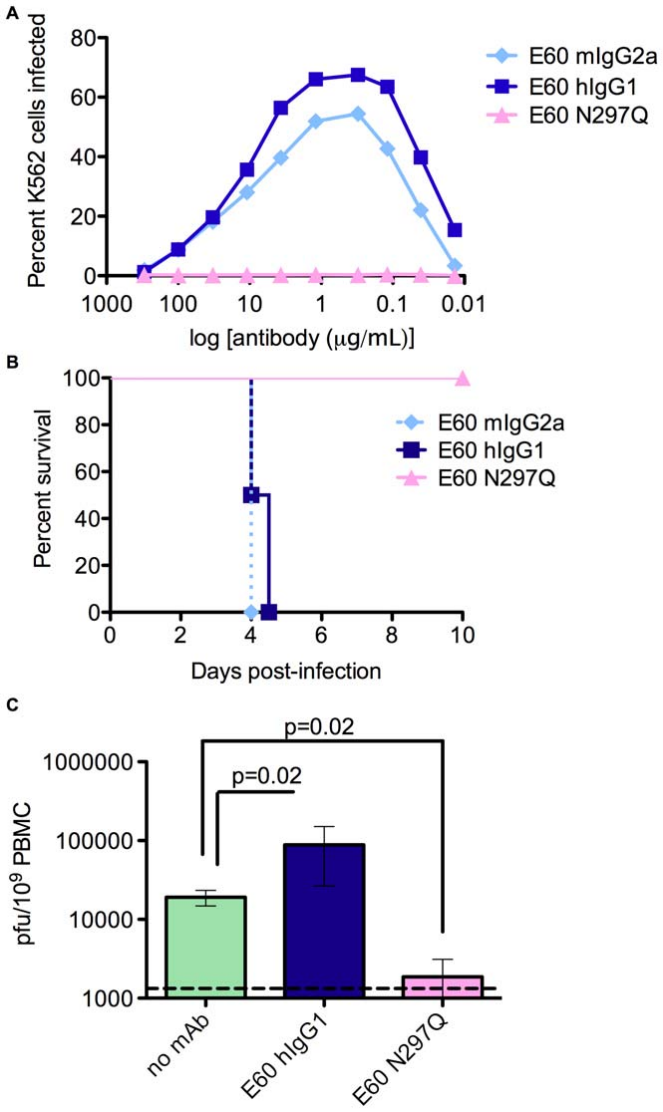
Antibodies with a mutated FcγR binding site cannot enhance DV infection *in vitro* or *in vivo*. **A.** Infection of K562 cells by DV2 in the presence of E60-mIgG2a, E60-hIgG1, E60-N297Q. **B.** Mice were administered 20 µg of the indicated E60 mAbs, challenged 24 hours later with 10^6^ pfu DV2 iv, and monitored for survival (n = 4 mice per group). p = 0.02 for E60-hIgG1 versus E60-N297Q. **C.** Mice were administered E60 mAbs and virus as in (B), and viral burden in peripheral blood cells was measured by plaque assay. n = 4 mice per group.

We followed up these studies using a mAb genetically engineered to eliminate FcγR binding. MAb E60 is a flavivirus cross-reactive neutralizing mouse IgG2a antibody that, similar to 4G2, binds to an epitope in the fusion peptide of domain II on the E protein [24],[25]. This mAb was cloned and the constant regions replaced [26] with those from human IgG1 to create an E60-chimeric human IgG1 (E60-hIgG1). In addition, an asparagine to glutamine variant at position 297 in human IgG1 was engineered (E60-N297Q), as this mutation abolishes FcγR binding without altering the half-life of the antibody in mouse serum [27]. Affinity measurements conducted by surface plasmon resonance indicated that E60-mouse IgG2a (E60-mIgG2a), E60-hIgG1, and E60-N297Q all exhibited similar binding to purified E protein (**Figure S3A**) and DV2-infected cells by flow cytometry (data not shown), as well as similar neutralizing activity against DV2 by both PRNT_50_ assay (0.23, 0.25, and 0.42 µg/ml, respectively) and a neutralization assay using DC-SIGN-expressing human target cells (**Figure S3B**). However, as expected, E60-mIgG2a and E60-hIgG1 enhanced DV2 infection of K562 cells *in vitro* whereas E60-N297Q did not (**Figure 6A**).

To test the ability of the E60-N297Q variant to enhance DV infection *in vivo*, mice were administered 20 µg of E60-mIgG2a, E60-hIgG1, and E60-N297Q 24 hours prior to infection with 10^6^ pfu of D2S10. Whereas both E60-mIgG2a and E60-hIgG1 consistently caused antibody-dependent mortality 4 to 5 days post-infection, equivalent doses of E60-N297Q caused neither morbidity nor mortality (**Figure 6B**). Instead, viremia and tissue viral burden in E60-N297Q recipients were substantially reduced, demonstrating that the N297Q mutation converted the *in vivo* effect of E60 on viral burden from enhancement to neutralization (**Figure 6C**, and data not shown).

The N297Q mutation also abolishes binding to complement component C1q [27]. Consequently, we generated a second variant antibody, E60-A330L, to assess whether the loss of C1q binding or the loss of FcγR binding explained the inability of E60-N297Q to mediate ADE. E60-A330L does not bind C1q but retains binding to FcγR [28], and we confirmed this by surface plasmon resonance (data not shown). E60-A330L exhibited similar binding and neutralization activity to E60-hIgG1, enhanced DV infection *in vitro* in K562 cells, and lethally enhanced a DV2-D2S10 infection *in vivo* (**Figure S3B, C, and D**, and data not shown). Thus, C1q interaction was not required for ADE *in vitro* or *in vivo*, and a loss of C1q binding does not explain the inability of E60-N297Q to enhance DV infection.

### An antibody that cannot bind FcγR has both prophylactic and therapeutic potential

**Figure 7:**
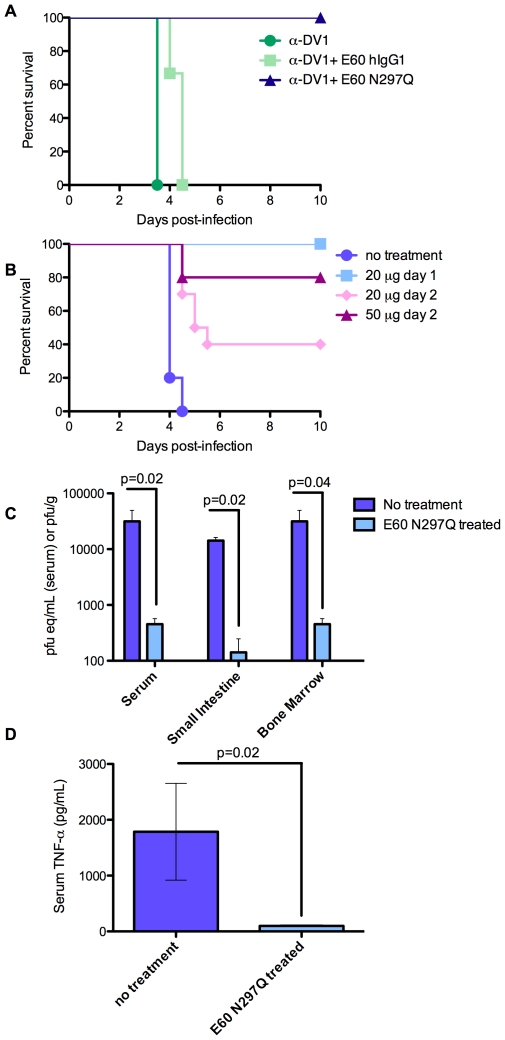
Antibodies with a mutated FcγR binding site have both prophylactic and therapeutic potential and reduce viral load and serum TNF-α in DV-infected mice. **A.** Mice were simultaneously administered 25 µl anti-DV1 serum and 20 µg of the indicated E60 mAbs ip, challenged 24 hours later with 2×10^5^ pfu of DV2 iv, and monitored for survival (n = 5 mice per group). p = 0.009 for E60-hIgG1 versus E60-N297Q recipients. **B.** Mice were administered 25 µl anti-DV1 serum ip, challenged 24 hours later with 10^5^ pfu of DV2 iv, treated by iv administration of E60-N297Q at the indicated doses and days post-infection, and monitored for survival. p-values compared to untreated controls (n = 9) were: p = 0.008 for 20 µg E60-N297Q on day 1 post-infection (n = 5); p = 0.005 for 20 µg E60-N297Q on day 2 post-infection (n = 10); and p = 0.02 for 50 µg E60-N297Q on day 2 post-infection (n = 5). Survival differences were compared using logrank tests. **C.** Mice were administered 25 µl of anti-DV1 serum ip and infected iv the next day with 10^5^ pfu of DV2. Mice were injected iv with either PBS (untreated) or 20 µg E60-N297Q 24 hours later. On day 3.5 p.i, mice were euthanized and serum and tissues were collected. Viral burden in serum, small intestine, and bone marrow were measured by qRT-PCR for serum and plaque assay for solid tissues. **D.** Serum TNF-α was measured by ELISA. n = 4 mice per group in all analyses. For viral load and TNF-α values, error bars represent standard deviations, and pairwise comparisons were performed by two-sided Wilcoxon rank sum tests.

The experiments above suggested that an N297Q variant antibody against DV could have potential as an antiviral intervention. To assess this, 20 µg of E60-hIgG1 or E60-N297Q was administered concurrently with 25 µl of anti-DV1 serum 1 day prior to infection with DV2. E60-N297Q protected mice against any signs of illness, whereas all recipients of anti-DV1/E60-hIgG1 succumbed to infection (**Figure 7A**). Post-exposure therapeutic application of E60-N297Q was evaluated by administering 25 µl anti-DV1 serum to mice, followed by infection with DV2 the next day, and iv administration of E60-N297Q or E60-hIgG1 on day 1 or 2 post-infection. While all mice treated with E60-hIgG1 succumbed to infection, lethality was completely prevented by a single 20 µg dose of E60-N297Q on day 1 (**Figure 7B** and data not shown), and E60-N297Q treatment significantly decreased viremia, tissue viral burden, and serum TNF-α levels as measured 3.5 days post-infection (**Figure 7C and 7D**, *p*<0.04). Moreover, 20 or 50 µg doses of E60-N297Q administered on day 2 resulted in 40% and 80% survival, respectively, demonstrating therapeutic efficacy for this antibody in mice (**Figure 7B**).

## Discussion

Understanding the immunopathogenesis of DV infection has been severely hampered by the lack of a small animal disease model. Thus, studies of ADE have been limited to epidemiological observations and *in vitro* experimentation. Here, we present the first model of antibody-enhanced lethal dengue disease *in vivo*. This work describes a long-sought mouse model for investigation of dengue pathogenesis, characterizes a clinically important mechanism of immunopathogenesis, has implications for vaccine development, and identifies a possible antibody-based antiviral strategy to treat life-threatening DV infection.

Numerous attempts have been made to establish a mouse model of dengue disease and have been limited by the relatively low susceptibility of mice to DV infection. Previous models have included intracerebral inoculation of DV or injection of very high (>10^9^ PFU) doses of virus into immunocompetent mice [29],[30]; infection of SCID [31]–[34] or NOD/SCID or RAG2γ (c)^−/−^mice [35],[36] implanted with human cells or cell lines; and use of various immunodeficient strains of mice [37],[38]. The most common outcome is neurovirulent disease, with a few recent exceptions [35],[36]. Of these, the AG129 mouse model has proven both useful and tractable, as it is permissive to infection with all four DV serotypes, displays relevant tissue and cellular tropism, produces long-lasting serotype-specific and serotype-cross-reactive anti-DV antibodies of a balanced isotype ratio, and generates a vascular leakage syndrome that in many respects resembles human dengue disease [9]–[11],[13],[12]. Nonetheless, we acknowledge that the lack of IFN receptors limits reproduction of some facets of human disease, especially in relation to cytokine profiles or infection conditions that are modulated by IFNs. However, the many similarities with specific features of human DV infection and the critical role for FcγR in ADE *in vivo* that we demonstrate here support the use of the AG129 model for specific avenues of dengue research. Interestingly, IFN-receptor deficient mice (A129) have recently been successfully adapted for other mosquito-borne viruses, including both Chikungunya and Yellow Fever [39],[40].

*In vivo* ADE models have also been established for other viruses, including Yellow Fever Virus (YFV), Murray Valley Encephalitis Virus (MVEV), Japanese Encephalitis Virus (JEV), and Feline Infectious Peritonitis Virus (FIPV) [41]–[46], in which passive transfer of antibody increases viral titers and/or mortality. These models show several parallels with our model of antibody-enhanced DENV infection: with FIPV, immune sera are able to enhance macrophage infection and disease during subsequent challenge with the same FIPV serotype in kittens [41],[46], and with MVEV, JEV, and YFV, enhanced mortality was observed in mice administered flavivirus cross-reactive polyclonal antibodies or non-neutralizing YFV-specific monoclonal antibodies [42]–[45]. However, none of these pathogens are associated with antibody-enhanced disease in humans. By modelling ADE with a pathogen known to cause antibody-enhanced disease in humans and using a model that displays a variety of relevant disease phenotypes, this report extends previous work on ADE to the ability to model human disease parameters and aid in the development of therapeutics.

*In vivo* evidence of ADE of DV infection was first described by Halstead *et al* [7] following the passive transfer of antibodies in the rhesus macaque. Similar data was recently obtained by Gonçalvez *et al* [8], where passive transfer of the serotype-cross-reactive mAb 1A5 enhanced DV4 viremia over a ∼30-fold concentration range (0.22–6 mg/kg). While both of these studies described elevated viremia, neither resulted in a clinical phenotype with parallels to human disease. Here, we describe enhancement of a mouse-adapted strain of DV2 by serotype-specific and cross-reactive sera as well as by monoclonal antibodies. Importantly, characterization of antibody-dependent dengue disease in the AG129 mouse maintains several parallels with severe disease in humans. Hallmark features of human DHF/DSS are vascular leak, higher viral burden, increased levels of serum cytokines such as TNF-α and IL-10, and platelet depletion [47]. All of these features were observed in our mouse model of ADE. Moreover, the magnitude of DV enhancement also mimics that seen in humans and non-human primates. We observed a 20-fold increase in viremia triggered by ADE; DV viremia in humans is reported to be 10- to 100-fold higher in DHF cases than in DF cases [48],[49], and ADE in macaques increases viremia 5–100 fold [7],[8]. Interestingly, in all of the disease parameters we examined, there was no apparent difference between lethality resulting from antibody-enhanced infection with a sublethal viral dose and lethality resulting from direct inoculation with a 100-fold higher viral dose. Thus, this model did not reveal any fundamental difference in the mechanisms of pathogenesis between antibody-enhanced and non-enhanced infection; rather, lethality here appears to be a result of higher viral burden, regardless of how such a burden was achieved.

To ensure that enhanced disease in the AG129 model was not solely a feature of the mouse-adapted strain, mice were infected with clinical isolates DV1 Western Pacific-74 and DV2 TSV01 in the presence of anti-DV antibodies, and enhanced viremia was observed in both cases. The lack of mortality in infections with these viruses is likely a result of the lower viral burden established by non-adapted strains even in the presence of enhancing antibody. Interestingly, mild fluid accumulation was also observed in the gastrointestinal organs in a subset of mice experiencing enhanced infection of non-adapted DV. As only a small fraction (0.5%) of human secondary DV infections results in severe disease, and some DV strains are more virulent than others based on genetic differences [50], the observed spectrum in disease severity is not surprising, but rather parallels the human condition.

Immunohistochemical (IHC) characterization of the cellular tropism associated with ADE using NS3-specific antibodies indicated infection in cells with morphology consistent with dendritic cells and tissue macrophages in the lymph node, small intestine, large intestine and bone marrow. Further characterization by flow cytometry supported the IHC data and demonstrated infection, as evidenced by both anti-E and anti-NS3 staining, in cells with surface markers of monocytes and macrophages in the bone marrow and sinusoidal endothelial cells in the liver. By both methodologies, the infected cell types identified in the murine model agree with those cells defined as the natural targets of DV in the human host [12],[18]. Interestingly, the infected cell types did not change between an enhanced and non-enhanced DV infection; rather, quantification by both IHC and flow cytometry indicated an increase in the number of infected cells. Taken together, antibody-enhanced disease appears to result in increased infection in the natural targets of DV infection and resulting pathogenesis that does not significantly differ from the disease that results when a 100-fold higher dose of DV is used in the absence of enhancing antibody.

In human infants who have acquired maternal anti-DV antibody, severe dengue can occur even when calculated neutralizing antibody titers against the secondary infecting serotype are >1∶100 [51]. Similarly, children with detectable neutralizing antibody against the infecting virus strain can develop DHF during secondary DV infections [22]. These studies indicate that the *in vitro* neutralization assay using BHK21 cells is not a consistent correlate of protection in humans. Similarly, our PRNT_50_ assays performed on serum samples from mice after antibody transfer but before virus challenge demonstrated that despite *in vitro* neutralizing activity at the time of infection, anti-DV1 sera, anti-DV2 sera, and 4G2 all enhanced infection *in vivo*. Enhanced disease was consistently observed in antibody-recipient mice with pre-infection neutralizing titers of <1∶200, but not greater. Thus, substantial neutralizing antibody levels appear to be required to prevent severe disease in this model. Of note, the passive transfer and primary infection scheme used does not examine anamnestic B and T cell immune responses, and thus, more accurately models DHF/DSS in infants with primary DV infection rather than secondary DV infections.

*In vitro* evidence had previously indicated that an interaction between the Fc portion of the antibody and the FcγR was necessary for ADE [8]; however, this hypothesis had never been corroborated *in vivo*. Using two different reagents – F(ab)′2 fragments of 4G2 and the N297Q variant of hE60-IgG1, we demonstrate that binding of the Fc portion of the antibody to the FcγR is required for ADE-induced disease. Further analysis with F(ab)′2 or the N297Q variant showed a reduction in viral titer below the level in mice receiving PBS in place of mAb. Thus, under conditions where the antibody cannot bind the FcγR, the F(ab) portion of the antibody can neutralize infection. These data also demonstrate that antibodies directed to the fusion loop in E domain II are capable of neutralizing DV infection independently from effector functions mediated by FcγR and C1q. Because the N297Q mutation also ablated the C1q binding site, we tested a second hE60 variant, hE60-A330L, that contained a mutation disrupting the complement C1q receptor binding site, but not the FcγR interaction. Mice receiving the hE60-A330L variant succumbed to an enhanced DV infection. This confirms that interaction of the anti-DENV mAb with the FcγR, and not binding of C1q, is essential for ADE *in vivo*.

Given the promising data with the hE60-N297Q variant, we tested the prophylactic and therapeutic efficacy of this antibody. When given as prophylaxis together with an enhancing amount of anti-DV1 serum, hE60-N297Q was completely protective. Although interesting, a DENV prophylactic is not likely to be a clinically useful reagent. However, when given 24 hours after an enhanced DENV infection, E60-N297Q completely protected against mortality; likewise, tissue viral load and systemic TNF-α levels in these mice at 3.5 days post-infection were significantly reduced. Two different doses of E60-N297Q, 20 and 50 µg, were administered 48 hours post-infection and resulted in 50% and 80% survival, respectively. Given the condensed timeframe of DENV pathogenesis in the AG129 model, E60-N297Q or similar therapeutic mAbs may have a broader time window for intervention and efficacy in humans or other animal models that display more protracted kinetics of DV infection.

In summary, we report the first animal model of lethal antibody-mediated enhancement of DV infection, describe virologic and pathologic changes induced by ADE, and define antibody conditions for protection and ADE in passive antibody transfer recipients. Furthermore, we show that antibodies engineered to prevent FcγR interaction exhibit prophylactic and therapeutic efficacy against DV infection, and thus have potential as a novel antiviral strategy against DV.

## Materials and Methods

### Ethics statement

All experimental procedures were pre-approved by the UC Berkeley Animal Care and Use Committee and were performed according to the guidelines of the UC Berkeley Animal Care and Use Committee.

### Viruses, cell lines and monoclonal antibodies

DV was propagated in the *Aedes albopictus* cell line C6/36 (American Type Culture Collection [ATCC]) as described elsewhere [52]. DV2 strain D2S10 (passaged 4 times in C6/36 cells) was derived in our laboratory [9] from the parental DV2 PL046 Taiwanese isolate as previously described [9]. The DV1 strain 98J was isolated in our laboratory from a patient from Guyana in 1998 [53] and passaged 7 times in C6/36 cells. The DV1 strain Western Pacific 74, originally isolated in Nauru in 1974, was obtained from the National Institutes for Biological Standards and Control (Hertfordshire, UK) and passaged 3 times in C6/36 cells. The DV2 strain TSV01, isolated in Townsville, Australia, in 1993 was obtained from W. Schul, passaged ∼10 times in C6/36 cells (Novartis Institute for Tropical Diseases, Singapore) [11]. Virus titers were obtained by plaque assay on baby hamster kidney cells (BHK21, clone 15) as described [52]. For mouse experiments, virus was concentrated by centrifugation at 53,000×g for 2 hours at 4°C and resuspended in cold PBS with 20% FBS (HyClone, Thermo Scientific). U937 DC-SIGN cells were obtained from A. de Silva (University of North Carolina, Chapel Hill) and grown in RPMI media (Invitrogen) at 37°C in 5% CO_2_. K562 cells were used for all enhancement assays and grown in RPMI media (Invitrogen) at 37°C in 5% CO_2_. The hybridoma of mAb 4G2 was purchased from ATCC, grown in serum-free medium (Invitrogen), and purified using protein G affinity chromatography (Thermo Scientific). Mouse mAb E60 and human E60-IgG1 (hE60), were obtained from M. Diamond, and hE60-N297Q was obtained from S. Johnson (MacroGenics, Inc.). The mouse E60 IgG2a mAb was originally generated against WNV E protein, reacts with an epitope in the fusion peptide in domain II, and cross-reacts with DV E proteins [25]. The generation of a chimeric human-mouse E60 with the human IgG1 constant regions and the mouse VH and VL was performed as described previously [26]. Point mutations in the Fc region that abolish FcγR and C1q binding (N297Q) or C1q binding alone (A330L) were introduced by QuikChange mutagenesis (Stratagene). All recombinant antibodies were produced after transfection of HEK-293T cells, harvesting of supernatant, and purification by protein A affinity chromatography.

### Infection of AG129 mice

AG129 mice [54] were originally obtained from M. Aguet (Swiss Institute for Experimental Cancer Research, Epalinges, Switzerland) and were bred in the University of California (UC) Berkeley Animal Facility. All experimental procedures were pre-approved and were performed according to the guidelines of the UC Berkeley Animal Care and Use Committee.

#### Generation of mouse anti-DV sera

Six to eight week-old AG129 mice were infected subcutaneously with 10^5^ pfu of either DV1 strain 98J or DV2 strain PL046 or PBS. Mice were sacrificed by terminal bleed six to eight weeks after infection. Serum was separated from whole blood by centrifugation, heat-inactivated, and frozen at −80°C.

#### Enhancement of DENV in vivo

Mice were injected intraperitoneally with mAb, PBS, or anti-DV sera in a total volume of 400 µl, then infected 20–24 hours later with DV by iv injection into the tail vein in a total volume of 100 µl. In some experiments, 50–100 µl of blood was obtained via retro-orbital bleed approximately 18 hours post-transfer (i.e., 4–6 hours prior to DV infection) and processed into serum as above.

### Measurement of cytokines and platelet counts

Cytokines were measured using commercially available ELISA kits (EBioscience). Platelet counts were obtained by diluting 20 µl of anticoagulated blood into Unopette reservoirs (BD) and counting on a hemocytometer.

### Quantitation of virus in tissues by plaque assay

Viral load was determined in the indicated tissues as previously described [52], and expressed as either pfu/g (all solid tissues) or pfu/10^9^ cells (bone marrow and PBMCs). To obtain PBMCs, 200–300 µl of whole blood was collected into EDTA-coated microtainer tubes (Becton Dickinson) after cardiac puncture. Samples were washed 3 times in red blood cell lysis buffer (eBioscience) and once in cold PBS, and resuspended in 250 µl alpha-MEM with 5% fetal bovine serum (FBS, Hyclone), 10 mM Hepes (Invitrogen) and 100 U penicillin/100 µg streptomycin (P/S; Invitrogen).

### Quantitation of virus in serum by quantitative RT-PCR

Viral RNA was extracted from 60 µl serum aliquots using Qia-Amp Viral RNA recovery kit (Qiagen). Quantitation of viral RNA utilized Taqman reagents (One Step RT-PCR Kit, Applied Biosystems, Foster City, CA) and an ABI PRISM 7700 sequence detection system as described [55]. Viremia is expressed as plaque-forming unit equivalents/ml, which was calculated by dividing the genomic RNA copy number in each sample by the genome:pfu ratio of C6/36-derived virus as determined by plaque assay and qRT-PCR.

### Immunohistochemistry

Tissues were collected at day 3.5 (n = 3–6 mice per group), formalin-fixed, and processed into paraffin sections. Serial sections from each tissue were stained for NS3 using MAb E1D8 or an isotype control as previously described [12]. For quantification of NS3^+^ cells, at least ten visual fields were counted for each sample except bone marrow, where four fields from four independent sections were counted due to the small area of mouse bone cross-sections. All pairwise comparisons were performed by two-sided Wilcoxon Rank Sum tests.

### Flow cytometry

***Bone marrow aspirates*** were collected by perfusing two femurs with cold, complete RPMI media (Invitrogen) containing 10% FBS (Hyclone), 10 mM Hepes (Invitrogen) and 100 U penicillin/100 µg streptomycin (P/S; Invitrogen). Resuspended cells were washed once in red cell lysis buffer and once in D-PBS (Invitrogen). The cells were subsequently resuspended in flow cytometry buffer containing D-PBS, 2.0% bovine serum albumin (BSA; Fisher Scientific) and 0.02% sodium azide (Sigma-Aldrich) and plated in a 96-well U-bottom plate (Becton-Dickinson) at 1×10^6^ cells/well. Cells were blocked with 5% normal rat serum (Jackson Laboratories) diluted in flow cytometry buffer. Bone marrow cells were stained extracellularly using CD11b-PeCy7 (eBioscience), CD11c-PE (eBioscience), and F4/80-TC (Caltag) or isotype control, and then fixed in 2% paraformaldehyde (Ted Pella, Inc.), washed and permeabilized with 0.1% saponin (Sigma-Aldrich). Intracellular staining was then performed with either 1) human anti-DV E mAb 87.1 (F. Sallusto and A. Lanzavecchia, Institute for Research in Biomedicine, Bellinzona, Switzerland) or isotype control mAb hIgG1 WNV-E16 (M.S. Diamond) followed by secondary goat anti-human IgG conjugated to Alexa488 (Invitrogen) or 2) mouse anti-NS3 mAb E1D8 conjugated to Alexa488 (Invitrogen) or isotype control (mIgG2a-Alexa488 (Invitrogen)). ***Livers*** were harvested into 10 mL cold, complete RPMI media and subsequently digested using 20 mg/mL collagenase VII (Sigma-Aldrich), washed, and the digested tissue passed over a 70-µM cell strainer (Fisher). The resulting cells were centrifuged over an Optiprep gradient (14.7%/22.2%), washed once with D-PBS, and plated in a 96-well plate at 1×10^6^ cells/well. The cells were stained extracellularly using CD31-PE (eBioscience), fixed in 2% PFA, permeabilized with 0.1% saponin, and stained intracellularly with either anti-E or anti-NS3 mAbs or isotype control as above. Data was collected using either an LSR II or FC-500 flow cytometer (Becton-Dickinson) and analyzed using FlowJo v8.8.6 software (TreeStar).

### Surface plasmon resonance

Monoclonal antibodies at a concentration range of 12.5 to 200 nM were injected over the surface of a Biacore 3000 instrument with immobilized E protein (∼300 RU) at a flow rate of 30 µl/min for 120 seconds and a dissociation time of 180 seconds. Binding curves at concentration zero were subtracted as blank. Kinetic parameters were calculated by fitting binding curves to a bivalent analyte binding model. The kinetic parameters were similar for binding of both mAb variants to E protein, as the difference between affinities is less than two-fold.

### Preparation of F(ab)′2 fragments and ELISA

4G2 F(ab)′2 fragments were generated using the F(ab)′2 Preparation kit (Pierce) according to the manufacturer's instructions. To ensure that the F(ab)′2 fragments did not contain residual Fc portions, the 4G2 F(ab)′2 proteins were diluted in SDS-PAGE loading dye, boiled, and electrophoresed on a 10–20% Tris-glycine gel (Bio Rad) and stained with Colloidal Blue (Invitrogen) overnight. To measure the stability of F(ab)′2 fragments *in vivo*, sera from mice given different amounts of F(ab)′2 were tested by ELISA for DV2 E protein binding. In brief, ELISA plates (Fisher Scientific) were coated with 2 µg/ml of recombinant DV2 E protein (Hawaii Biotech Inc.) in carbonate coating buffer, pH 9.6 overnight at 4°C. The plate was blocked for 1 hour at room temperature in 5% nonfat dry milk and 5% donkey serum (Jackson Laboratories) in PBS-0.5% Tween 20. After washing, 50 µl of serum containing intact 4G2 or F(ab)′2 4G2 diluted 1∶10 in blocking buffer was added to the plates. After washing, 100 µl of either goat anti-mouse anti-F(ab)′2 (Jackson Laboratories) or goat anti-mouse anti-Fc (Jackson Laboratories) diluted 1∶1000 in PBS-T was added as secondary antibody. Biotinylated mouse anti-goat antibody (Jackson Laboratories) was added as a tertiary antibody, followed by streptavidin-alkaline phosphatase (Zymed). P-Nitrophenyl phosphate (PnPP; Sigma Aldrich) was added as the substrate, and the reaction was stopped with 3M NaOH and read in an EL_X_-808 ultra microplate reader (Bio-Tek Instruments) at 405 nm.

### Neutralization assay using U937 DC-SIGN cells and plaque reduction neutralization test

Serial 3-fold dilutions of antibodies were mixed with DV2 D2S10 virus at a multiplicity of infection (MOI) generating 7–15% infection of U937 DC-SIGN cells in a 96-well U bottom plate as described previously [14]. After infection for 24 hours, the cells were washed once with flow cytometry buffer and fixed in 2% PFA for 10 minutes at room temperature. The cells were then permeabilized in FACS buffer with 0.1% saponin (Sigma Aldrich) and stained with 2.5 µg/mL 4G2-Alexa 488 (Invitrogen). The cells were washed twice, and percent infection determined by flow cytometry on a Beckman Coulter EPICS XL flow cytometer. The resulting raw data was expressed in GraphPad Prism 5.0 software as percent infection versus log_10_ of the serum dilution, and a sigmoidal dose-response curve with a variable slope was applied to determine the antibody titer coinciding with a 50% reduction in infection as compared to the no-serum control (NT_50_). The plaque reduction neutralization test (PRNT) was performed in duplicate as described previously [13].

### *In vitro* measurement of ADE

Serial 3-fold dilutions of antibody were mixed with DV2 D2S10 virus in duplicate for 45 min at 37°C, then mixed with K562 cells at MOI of 1 for 48 hours [23] in a 96-well plate. The cells were subsequently washed once with FACS buffer and fixed in 2% PFA for 10 minutes at room temperature. To stain, the cells were permeabilized in FACS buffer with 0.1% saponin (Sigma Aldrich), and then stained with 2.5 µg/mL 4G2-Alexa 488 (Invitrogen). The cells were washed twice, and percent infection was determined by flow cytometry on a Beckman Coulter EPICS XL flow cytometer. The resulting data was expressed as percent cellular infection versus log_10_ of the serum dilution in Microsoft Windows Excel.

### Statistical analysis

Kaplan-Meier survival curves were used to display mortality data, and log rank analyses were used to determine statistical significance between experimental groups. Non-parametric analyses using the two-sided Wilcoxon rank sum tests were used for pairwise comparisons of viral load, cytokines, and platelet counts. A Fisher's exact test was used to examine survival on day 4 post-infection in F(ab′)2 experiments because the instability of F(ab′)2 fragments necessitated comparison at a single time point. Calculations were performed in GraphPad Prism 5.0 software.

## Supporting Information

Table S1 Effect of anti-DV1 serum and viral dose on morbidity and mortality
(0.05 MB PDF)

Table S2 Morbidity and mortality with 10^5^ pfu DV2 inoculation under varying antibody conditions
(0.06 MB PDF)

Figure S1 Phenotyping of DV-infected cell types in bone marrow and liver under non-ADE and ADE conditions. Mice were administered naïve serum (NMS) and 24 hours later injected iv with either PBS (uninfected) or 10^5^ DV2 D2S10 (non-ADE) or were infected with 10^5^ DV2 D2S10 24 hours after receiving anti-DV1 serum (ADE). Bone marrow aspirates and livers were collected on day 3.5 post-infection. (A) The bone marrow cells were stained and collected as described in Materials and Methods. The majority of DV^+^ cells were CD11b^+^ (65%); thus, cells were initially gated on CD11b (monocyte marker). The isotype control for CD11b is depicted in pink in the initial histogram. Scatterplots of CD11b^+^ cells stained with anti-DV E or isotype control and either CD11c (dendritic cell marker) or F4/80 (macrophage marker) are shown for one representative animal out of six. Similar results were obtained using anti-DV NS3 mAb E1D8: of CD11b^+^ cells, 0.33%, 0.96% and 3.03% were CD11c^+^NS3^+^ in uninfected, non-ADE, and ADE conditions, respectively; and 0.39%, 0.96%, and 3.34% were F4/80^+^NS3^+^ in uninfected, non-ADE, and ADE conditions, respectively. (B) Livers were processed and stained as described in Materials and Methods. Data collection and analysis was performed as in (A). Scatterplots of cells stained with CD31 (endothelial cell marker) and anti-DV NS3 or isotype control are shown for one representative animal out of six. Similar results were obtained with human anti-DV E.
(1.45 MB TIF)

Figure S2 Characterization of F(ab′)2 fragments of 4G2. MAb 4G2 was processed into F(ab′)2 fragments using the Immunopure F(ab′)2 kit (Pierce). (A) Intact 4G2 and purified F(ab′)2 fragments were tested for reactivity against purified DV2 E protein (Hawaii Biotech Inc.) by ELISA and detected with anti-F(ab′)2-specific antibody. (B) ELISA was performed as in (A), but with detection antibody specific for the Fc portion of mouse IgG. (C) Mice were administered 4G2 ip at doses shown to enhance infection *in vivo* (5 or 80 µg), and serum was collected 24 hours later. 4G2 F(ab′)2 fragments were administered to mice iv and serum collected 1 and 24 hours later. Serum levels of intact 4G2 and F(ab′)2 fragment were measured by reactivity to DV2 E protein by ELISA using anti-F(ab′)2-specific antibody.
(0.37 MB PDF)

Figure S3 Further characterizations of E60 antibody variants. (A) Monoclonal antibodies at a concentration range of 12.5–200 nM were injected over the surface of a Biacore 3000 with immobilized E protein (∼300RU) at a flow rate of 30 ml/min for 120 sec and a dissociation time of 180 sec. Binding curves at concentration zero were subtracted as blank. Kinetic parameters were calculated by fitting binding curves to a bivalent analyte binding model. The kinetic parameters are similar for binding of both mAb variants to E protein, as the difference between affinities is less than two-fold. (B) Neutralizing activity of E60 variants on DC-SIGN-expressing U937 cells. DV2 was incubated with the indicated concentrations of each E60 variant MAb, applied to U937 cells expressing the DV attachment receptor DC-SIGN, and the percentage of cells infected with DV was assessed 24 hours later by flow cytometry staining with Alexa488-labelled anti-DV E protein MAb. (C) E60 A330L enhances DV infection *in vitro*. Enhancement assays were performed as in Figure 6A using E60-A330L. E60-N297Q is shown for comparison. D. E60 A330L enhances DV infection *in vivo*. Mice were administered 20 µg E60-A330L or PBS (no MAb) ip, then infected 24 hours later with 10^6^ pfu DV2 iv. Survival was monitored for 10 days. n = 4 for E60-A330L recipients and n = 6 for no MAb controls; p = 0.014 by logrank test.
(0.68 MB PDF)
