# Supplementary material for: Lethal Antibody Enhancement of Dengue Disease in Mice Is Prevented by Fc Modification
Source: PLoS Pathog. 2010 Feb 12;6(2):e1000790. doi: 10.1371/journal.ppat.1000790 (PMC2820409; doi:10.1371/journal.ppat.1000790)
Supplement: Table S1 — Effect of anti-DV1 serum and viral dose on morbidity and mortality (0.05 MB PDF) [file ppat.1000790.s001.pdf]

Table S1. Effect of anti-DV1 serum and viral dose on morbidity and mortality.

Serum[a]	DV2 dose (pfu)	Morbidity[b]	Morbidity p-value vs. NMS control[c]	Mortality	Mean survival time ± s.d. (days)[d]	Mortality p-value vs. NMS control[e]
**NMS[f]**	10^7	15/15		14/15	4.2 ± 0.4	
**NMS**	10^6	6/7		0/7	n.a.[g]	
**α-DV1[h]**	10^6	12/12	0.5	11/12	3.5 ± 0.3	0.003
**NMS**	10^5	0/11		0/11	n.a.	
**α-DV1**	10^5	4/4	0.03	4/4	4.0 ± 0.0	0.001
**NMS**	10^4	0/4		0/4	n.a.	
**α-DV1**	10^4	4/4	0.03	1/4	5.5	0.3

[a]100μl of the indicated antiserum was given to all mice in each group.

[b]Mice were scored as morbid if hunched posture, ruffled fur, and lethargy were simultaneously present at any time during the first ten days post-infection.

[c]Result of two-sided Fisher's exact test comparing α-DV1 recipients to NMS recipients at the same viral dose.

[d]Mean survival time of mice who succumbed to infection during 10-day timecourse.

[e]Result of logrank analysis comparing anti-DV1 recipients to NMS recipients at the same viral dose.

[f]Naive mouse serum.

[g]Not applicable. No mortality occurred in these groups.

[h]Anti-DV1 serum collected 8 weeks after infection of AG129 mice with 10^5 pfu DV1.
