# Supplementary material for: Lethal Antibody Enhancement of Dengue Disease in Mice Is Prevented by Fc Modification
Source: PLoS Pathog. 2010 Feb 12;6(2):e1000790. doi: 10.1371/journal.ppat.1000790 (PMC2820409; doi:10.1371/journal.ppat.1000790)
Supplement: Table S2 — Morbidity and mortality with 105 pfu DV2 inoculation under varying antibody conditions (0.06 MB PDF) [file ppat.1000790.s002.pdf]

Table S2. Morbidity and mortality with 10^5 pfu DV2 inoculation under varying antibody conditions.

Antiserum or antibody	Antiserum or antibody dose[a]	Morbidity[b]	Morbidity p-value vs. control[c]	Mortality	Mean survival time ± s.d. (days)[d]	Mortality p-value vs. NMS control[e]
**NMS[f]**	100	0/11		0/11	n.a.[g]	
**α-DV1[h]**	400	4/4	0.001	4/4	4.5 ± 0.0	0.001
**α-DV1**	100	4/4	0.001	4/4	4.0 ± 0.0	0.001
**α-DV1**	25	4/4	0.001	4/4	4.2 ± 0.3	0.001
**α-DV1**	6.25	4/4	0.001	2/4	5.0 ± 0.0	0.02
**α-DV1**	1.25	0/4	1.0	0/4	n.a.	1.0
**α-DV2[i]**	400	0/4	1.0	0/4	n.a.	1.0
**α-DV2**	100	4/4	0.001	4/4	4.0 ± 0.0	0.001
**α-DV2**	25	4/4	0.001	4/4	4.0 ± 0.0	0.001
**α-DV2**	6.25	4/4	0.001	4/4	4.0 ± 0.0	0.001
**α-DV2**	1.25	4/4	0.001	4/4	4.0 ± 0.0	0.001
**IgG2a**	20	0/8		0/8	n.a.	
**4G2**	400	0/7	1.0	0/7	n.a.	1.0
**4G2**	80	10/10	0.00002	9/10	4.4 ± 0.4	0.001
**4G2**	20	5/5	0.0008	5/5	4.0 ± 0.4	0.005
**4G2**	5	6/6	0.0003	6/6	4.4 ± 0.4	0.0005
**4G2**	1.25	5/6	0.003	3/6	4.8 ± 0.3	0.03

[a]Dose of antiserum administered in μl or dose of mAb administered in μg.

[b]Mice were scored as morbid if hunched posture, ruffled fur, and lethargy were simultaneously present at any time during the first ten days post-infection.

[c]Result of two-sided Fisher's exact test comparing antiserum recipients to NMS recipients, or 4G2 recipients to IgG2a recipients.

[d]Mean survival time of mice who succumbed to infection during 10-day timecourse.

[e]Result of logrank analysis comparing anti-DV1 recipients to NMS recipients at the same viral dose.

[f]Naive mouse serum.

[g]Not applicable. No mortality occurred in these groups.

[h]Anti-DV1 serum collected 8 weeks after infection of AG129 mice with 10^5 pfu DV1 strain 98J.

[i]Anti-DV2 serum collected 8 weeks after infection of AG129 mice with 10^5 pfu DV2 strain PL046.
