# Supplementary figures and images for: Lethal Antibody Enhancement of Dengue Disease in Mice Is Prevented by Fc Modification
Source: PLoS Pathog. 2010 Feb 12;6(2):e1000790. doi: 10.1371/journal.ppat.1000790 (PMC2820409; doi:10.1371/journal.ppat.1000790)

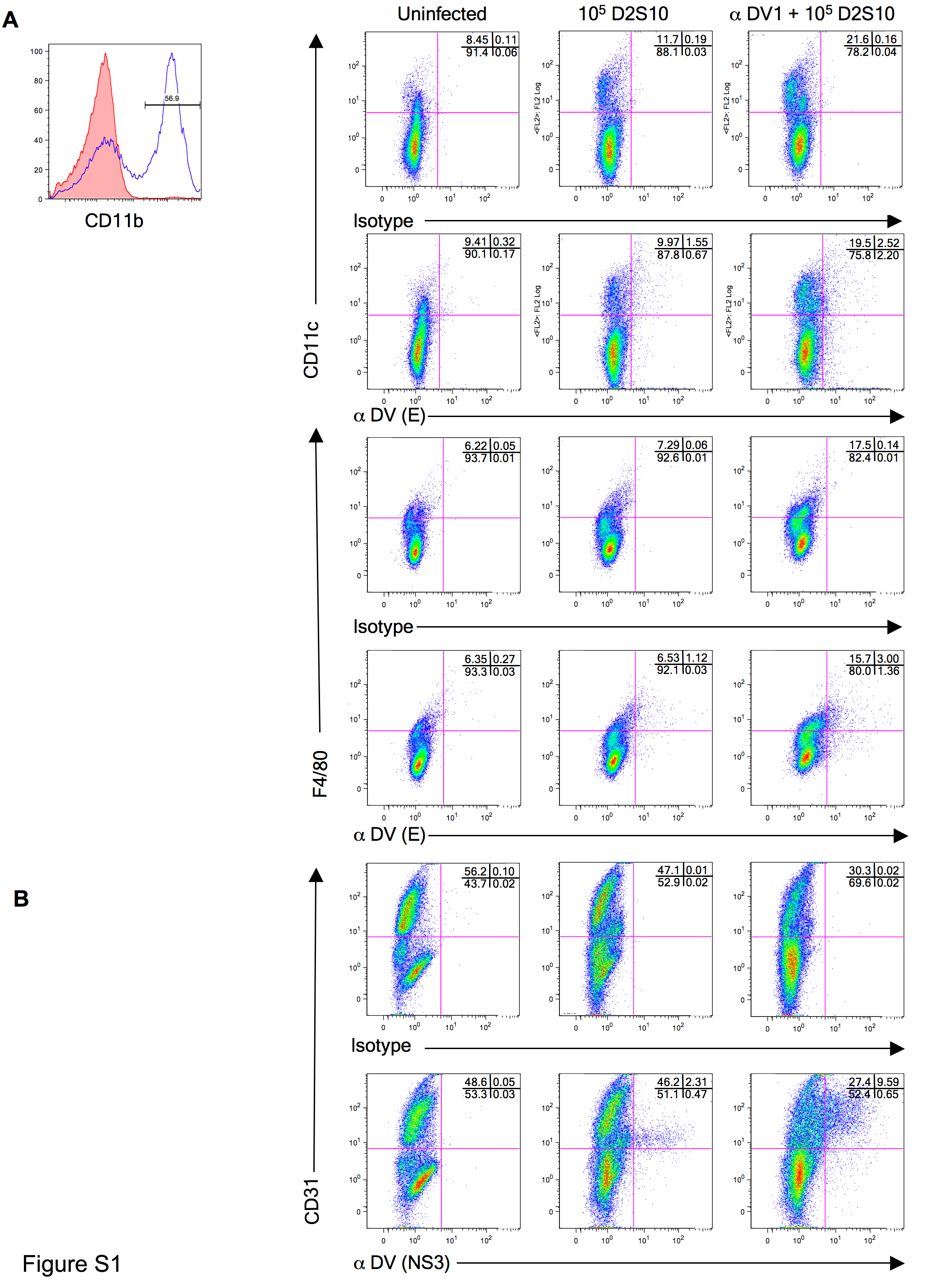

Supplement: Figure S1 — Phenotyping of DV-infected cell types in bone marrow and liver under non-ADE and ADE conditions. Mice were administered naïve serum (NMS) and 24 hours later injected iv with either PBS (uninfected) or 105 DV2 D2S10 (non-ADE) or were infected with 105 DV2 D2S10 24 hours after receiving anti-DV1 serum (ADE). Bone marrow aspirates and livers were collected on day 3.5 post-infection. (A) The bone marrow cells were stained and collected as described in Materials and Methods. The majority of DV+ cells were CD11b+ (65%); thus, cells were initially gated on CD11b (monocyte marker). The isotype control for CD11b is depicted in pink in the initial histogram. Scatterplots of CD11b+ cells stained with anti-DV E or isotype control and either CD11c (dendritic cell marker) or F4/80 (macrophage marker) are shown for one representative animal out of six. Similar results were obtained using anti-DV NS3 mAb E1D8: of CD11b+ cells, 0.33%, 0.96% and 3.03% were CD11c+NS3+ in uninfected, non-ADE, and ADE conditions, respectively; and 0.39%, 0.96%, and 3.34% were F4/80+NS3+ in uninfected, non-ADE, and ADE conditions, respectively. (B) Livers were processed and stained as described in Materials and Methods. Data collection and analysis was performed as in (A). Scatterplots of cells stained with CD31 (endothelial cell marker) and anti-DV NS3 or isotype control are shown for one representative animal out of six. Similar results were obtained with human anti-DV E. (1.45 MB TIF) [file ppat.1000790.s003.tif]

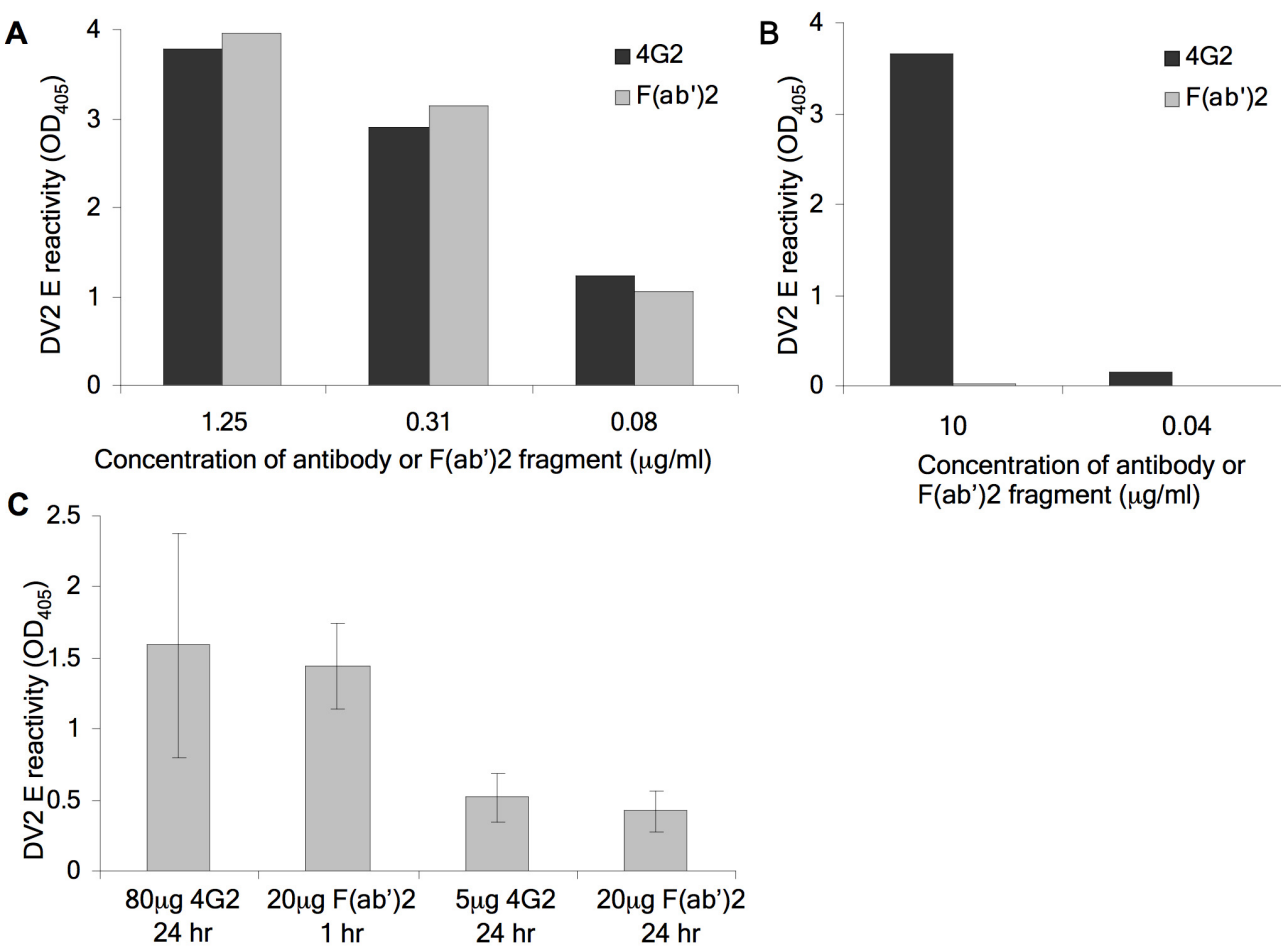

Figure S2

Supplement: Figure S2 — Characterization of F(ab′)2 fragments of 4G2. MAb 4G2 was processed into F(ab′)2 fragments using the Immunopure F(ab′)2 kit (Pierce). (A) Intact 4G2 and purified F(ab′)2 fragments were tested for reactivity against purified DV2 E protein (Hawaii Biotech Inc.) by ELISA and detected with anti-F(ab′)2-specific antibody. (B) ELISA was performed as in (A), but with detection antibody specific for the Fc portion of mouse IgG. (C) Mice were administered 4G2 ip at doses shown to enhance infection in vivo (5 or 80 µg), and serum was collected 24 hours later. 4G2 F(ab′)2 fragments were administered to mice iv and serum collected 1 and 24 hours later. Serum levels of intact 4G2 and F(ab′)2 fragment were measured by reactivity to DV2 E protein by ELISA using anti-F(ab′)2-specific antibody. (0.37 MB PDF) [file ppat.1000790.s004.pdf]

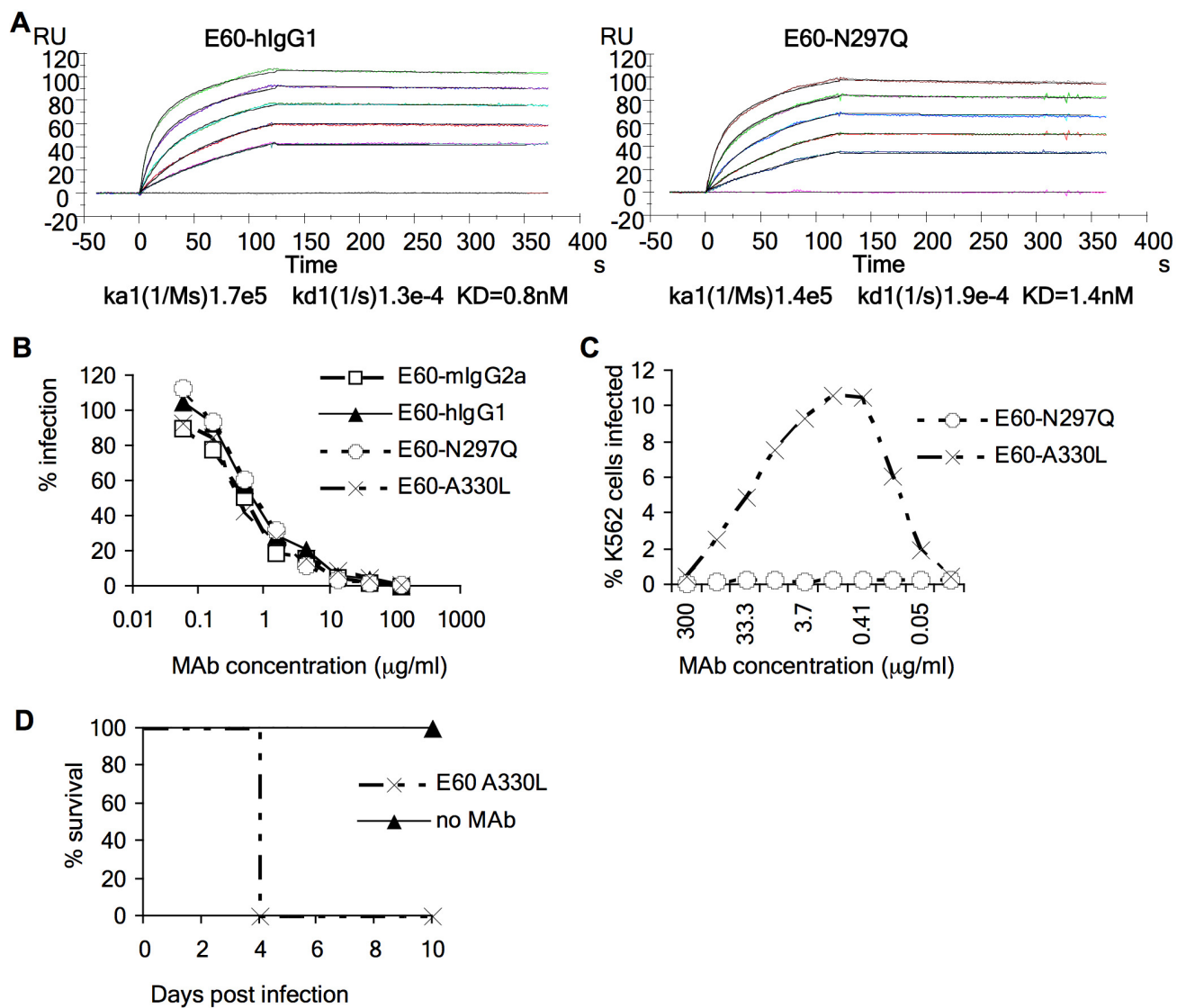

Figure S3

Supplement: Figure S3 — Further characterizations of E60 antibody variants. (A) Monoclonal antibodies at a concentration range of 12.5–200 nM were injected over the surface of a Biacore 3000 with immobilized E protein (∼300RU) at a flow rate of 30 ml/min for 120 sec and a dissociation time of 180 sec. Binding curves at concentration zero were subtracted as blank. Kinetic parameters were calculated by fitting binding curves to a bivalent analyte binding model. The kinetic parameters are similar for binding of both mAb variants to E protein, as the difference between affinities is less than two-fold. (B) Neutralizing activity of E60 variants on DC-SIGN-expressing U937 cells. DV2 was incubated with the indicated concentrations of each E60 variant MAb, applied to U937 cells expressing the DV attachment receptor DC-SIGN, and the percentage of cells infected with DV was assessed 24 hours later by flow cytometry staining with Alexa488-labelled anti-DV E protein MAb. (C) E60 A330L enhances DV infection in vitro. Enhancement assays were performed as in Figure 6A using E60-A330L. E60-N297Q is shown for comparison. D. E60 A330L enhances DV infection in vivo. Mice were administered 20 µg E60-A330L or PBS (no MAb) ip, then infected 24 hours later with 106 pfu DV2 iv. Survival was monitored for 10 days. n = 4 for E60-A330L recipients and n = 6 for no MAb controls; p = 0.014 by logrank test. (0.68 MB PDF) [file ppat.1000790.s005.pdf]
